# The impact of Patient and Public Involvement in the SlowMo study: Reflections on peer innovation

**DOI:** 10.1111/hex.13362

**Published:** 2021-09-28

**Authors:** Kathryn Greenwood, Sam Robertson, Evelin Vogel, Claire Vella, Thomas Ward, Alison McGourty, Cat Sacadura, Amy Hardy, Mar Rus‐Calafell, Nicola Collett, Richard Emsley, Daniel Freeman, David Fowler, Elizabeth Kuipers, Paul Bebbington, Graham Dunn, Philippa Garety, the SlowMo Patient and Public Involvement (PPI) team

**Affiliations:** ^1^ School of Psychology University of Sussex Brighton UK; ^2^ Sussex Partnership NHS Foundation Trust Worthing UK; ^3^ Department of Psychology, Institute of Psychiatry, Psychology and Neuroscience King's College London London UK; ^4^ South London and Maudsley NHS Foundation Trust London UK; ^5^ Oxford Health NHS Foundation Trust Oxford UK; ^6^ Department of Biostatistics and Health Informatics, Institute of Psychiatry, Psychology and Neuroscience King's College London London UK; ^7^ Department of Psychiatry Oxford University Oxford UK; ^8^ Division of Psychiatry University College London London UK; ^9^ Centre for Biostatistics, School of Health Sciences, Manchester Academic Health Science CentreManchester Academic Health Science Centre The University of Manchester Manchester UK

**Keywords:** Cognitive Behavioural Therapy, digital health, impact, mobile applications, paranoia, Patient and Public Involvement (PPI)

## Abstract

**Background:**

The SlowMo study demonstrated the effects of SlowMo, an eight‐session digitally supported reasoning intervention, on paranoia in a large‐scale randomized‐controlled trial with 362 participants with schizophrenia‐spectrum psychosis.

**Aim:**

The current evaluation aimed to investigate the impact of Patient and Public Involvement (PPI) in the SlowMo study.

**Method:**

PPI members were six women and three men from Sussex, Oxford and London with experience of using mental health services for psychosis. They received training and met at least 3‐monthly throughout the project. The impact of PPI was captured quantitatively and qualitatively through (i) a PPI log of recommendations and implementation; (ii) written subjective experiences of PPI members; (iii) meeting minutes; and (iv) outputs produced.

**Results:**

The PPI log revealed 107 recommendations arising from PPI meetings, of which 87 (81%) were implemented. Implementation was greater for recruitment‐, data collection‐ and organization‐related actions than for dissemination and emergent innovations. Qualitative feedback revealed impacts on study recruitment, data collection, PPI participants' confidence, knowledge, career aspirations and society more widely. Outputs produced included a film about psychosis that aired on BBC primetime television, novel webpages and journal articles. Barriers to PPI impact included geography, travel, funding, co‐ordination and well‐being.

**Discussion:**

A future challenge for PPI impact will be the extent to which peer innovation (innovative PPI‐led ideas) can be supported within research study delivery.

**Patient and Public Contribution:**

Planned Patient and Public Contribution in SlowMo comprised consultation and collaboration in (i) design, (ii) recruitment, (iii) qualitative interviews and analysis of service users' experiences of SlowMo therapy and (iv) dissemination.

## 1 BACKGROUND

Patient and Public Involvement (PPI) in research is increasingly important in the NHS, as it is proposed to enhance the value, credibility, effectiveness and ethical conduct of the research, and ensure that the research process and outcomes are patient‐centred. However, it is notoriously challenging to demonstrate the added value or the impact of PPI in research, and few studies report this impact. The current evaluation reports the impact of PPI in the SlowMo study.

### 1.1 The SlowMo study

The SlowMo study investigated the effects of SlowMo, an eight‐session digitally supported cognitive‐behavioural reasoning intervention, on paranoia and the mechanisms of change over 24 weeks in a large‐scale randomized‐controlled trial with 362 participants with schizophrenia‐spectrum psychosis and distressing, persistent paranoia across three UK sites (London, Sussex and Oxford).^1^ The intervention builds awareness of unhelpful ‘fast thinking’ and supports people to ‘Slow down for a Moment’ to find ways of feeling safer. Sessions are assisted by the SlowMo ‘webapp’, delivered via a touchscreen laptop, with interactive features including animated vignettes and personalized thought ‘bubbles’, and a mobile phone app that provides access to SlowMo strategies in daily life. The overall pattern of results clearly indicated that SlowMo was beneficial for paranoia, with 10/11 paranoia measures at 12 weeks and 8/11 at 24 weeks, demonstrating significant small‐moderate effects. Sustained moderate effects were found on all observer‐rated measures of persecutory delusions, and important improvements were also reported on self‐esteem, worry, well‐being and quality of life.

### 1.2 Definitions of PPI in research

The NIHR INVOLVE guidance (2020)^2^ on Patient and Public Involvement (PPI) defines PPI as ‘research being carried out “with” or “by” members of the public rather than “to”, “about” or “for” them’. Consultation is defined as one‐off or regular advice that may or may not be acted upon, whereas collaboration involves service users and researchers working in partnership with clearly agreed roles.

### 1.3 Theoretical rationale and influences

The theoretical rationale behind PPI in the SlowMo study was the expectation of epistemic improvements in the rigour, relevance and reach (three Rs) of the research.^3^ Indeed, there is growing evidence for the impact of PPI on the processes and outcomes of mental health research, through the increased reach of recruitment,^4^ relevance of dissemination that involves service users^5^, ^6^ and the enhanced rigour, openness and honesty of responses when service user participants are interviewed by their peers.^7^, ^8^, ^9^ The roles for PPI in the SlowMo study were thus focussed on support for recruitment, qualitative interview design and data collection and dissemination strategies. This identification of clear roles also served to minimize the risk of tokenism in the PPI contribution, wherein the absence of specific PPI aims leads to a self‐fulfilling prophecy of failure to demonstrate value and impact.^10^

Consistent with the epistemic framework for PPI, the study incorporated a consideration of these three Rs in the impact of PPI, and the PPI outcomes are reported in this paper, with reference to the GRIPP‐2 reporting checklist for PPI in research.^11^ The approach was influenced by the previous experiences of the PPI lead in collaborating with experts by experience, peer researchers and consultants^12^, ^13^, ^14^ and by the research team's interaction with service users in the development of the intervention and subsequent grant application, as outlined elsewhere.^15^, ^16^

### 1.4 Conceptual models and influences

Ives et al.^17^ differentiate between PPI that is ‘Consultation’, which is by invitation, top‐down, pragmatic and process oriented, focused on rigour, relevance and reach, and ‘Partnership‐Alliance’, or ‘Collaboration’, which is bottom‐up, rights‐based and process oriented, representing community values, joint decision‐making and the encouragement to offer new ideas. Consultation in the SlowMo study built on the ‘Critical Friend’ model, where a critical friend is a trusted person who asks provocative questions, provides data to be examined through another lens and offers a critique of a person's work as a friend. The friend is an advocate for the success of that work.^18^ The consultant role is thus objective and outside of the immediate research team.^17^ However, the SlowMo PPI approach also incorporated a ‘Collaborative, co‐produced’ model of peer researcher, wherein peer researchers ‘work collaboratively by drawing on their individual and collective expertise and knowledge to design and deliver the research study’.^19^ The peer researcher role included co‐design of methodology, data collection and analysis of the SlowMo study qualitative research, and in this respect, held some overlap with Ives et al.'s Partnership‐Alliance.^17^

### 1.5 PPI in the grant application phase

Before the current project, an extensive research programme incorporating both feasibility and an interactive human‐centred design approach was undertaken as outlined in Hardy et al.^15^, ^16^ Revisions were made to the name and design of the intervention, advice on pacing and personalizing the intervention led to an extension from six to eight sessions, language was made more accessible and the content was individualized.

PPI input for the current project commenced with the grant application. The PPI consultants influenced the choice of the primary outcome measure, which assessed distress and paranoia. They also advised that the intervention should address well‐being, functioning and distress, such that these were incorporated into the outcome measures, alongside a secondary outcome measure of self‐esteem. All the PPI consultants felt strongly that there was a need to improve treatments and access to treatments for distressing paranoia.

### 1.6 Lay versus expert PPI

One challenge in the identification of suitable PPI members lay in the well‐documented tension between the recruitment of lay service users, versus professionalized ‘expert’ PPI members,^17^ as a result of the incorporation of both lay consultant and peer researcher roles. Ives et al.^17^ propose a paradox. Lay PPI consultants may struggle to contribute meaningfully in peer researcher roles, involving research leadership, data collection or analysis, due to their lack of appropriate training. Yet, the provision of training required for collaborative peer research roles produces ‘expert’ service users with a track record of PPI, who may then no longer hold their original critical friend perspective, but instead share the language and perspective of the researcher. Staley^20^ argues that there are different levels of involvement requiring different levels of expertise and appropriately matched training. Consultation in relation to the recruitment of trial participants, for example, may be more valuable from lay service users, whilst qualitative data collection requires training and the development of expertise.^20^ In the SlowMo study, this tension was addressed though the recruitment of service users with a range of previous PPI expertise and by delivering training in 6‐monthly intervals focussed on different roles, which progressed from consultant to peer researcher as the project progressed.

### 1.7 Aims of PPI in the SlowMo study

The aims of PPI in the SlowMo study were that the PPI team would be involved in three specific aspects of work: (1) Assisting study recruitment, by presenting the research to teams and participants and giving their perspective on the study, and helping with the development of materials such as leaflets; (2) Designing and conducting qualitative interviews on participants' experiences of receiving SlowMo therapy; and (3) Assisting in the future dissemination of findings.

Funding was secured to provide for 8 hours of consultation per month on average throughout the duration of the project. To assist in meeting these aims, the PPI team received regular training and supervision, met as a group regionally and also project‐wide, and were invited to study management meetings.

The aim of the current evaluation was to investigate the impact of PPI in the SlowMo study.

## 2 METHOD

### 2.1 Identification of PPI members

PPI members for the SlowMo PPI teams were identified through a combination of (i) recruitment from pre‐existing PPI research and consultation groups, (ii) identification of people who had themselves taken part in the previous or current SlowMo research and (iii) direct expressions of interest, in response to publicity. The PPI teams comprised nine people: two women and one man in Sussex, two women in Oxford and two women and two men in London. They were aged between 30 and 56 years; one woman and two men (all from London) were from a BAME background, whilst all others were White British. All members had previous experiences of using mental health services for a psychosis spectrum condition. The principles of ethics were maintained including anonymity and confidentiality, and first names have been used with permission.

### 2.2 Methods through which PPI members were involved

**Figure 1:**
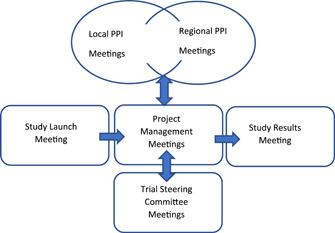
Interrelationship between SlowMo study and Patient and Public Involvement meetings

Involvement commenced with a whole PPI team introduction and training session, co‐facilitated by the study PPI lead (K. G.) and local site leads. This was followed by a second training 6 months later. Thereafter, regional teams met approximately every 1–3 months, with group discussion and activity facilitated by the respective site lead, and also by a designated Expert by Experience PPI lead at the Sussex site (S.R.). The PPI team together, made a plan to meet as a whole study group, once or twice per year. Finally, PPI members were invited to key study meetings including the study launch, study steering meetings and the results meeting (see Figure 1).

### 2.3 PPI induction and training

SlowMo PPI participants each received either an introductory session to the SlowMo therapy to demonstrate how the SlowMo intervention worked, or received a full course of SlowMo therapy, before commencing the PPI role.

A whole‐group introductory training programme was designed by the PPI lead (K. G.), based on previous training programmes that were co‐produced with service user involvement leads. The training focused on (i) an introduction to PPI and the ‘critical friend’ model, (ii) discussing, disclosing and using experiences, (iii) an introduction to research methods, PPI and peer research in the SlowMo study and (iv) supervision and safe‐guarding. Subsequent whole‐group training was more consultative and PPI‐led, and included (i) site updates, (ii) specific project work, (iii) role play practice and feedback in preparation for qualitative interview data collection and (iv) the development of personalized role boundaries, disclosure, keeping well and supervision plans. As recommended by Friesen et al.,^21^ the PPI training prioritized the development of service users' capacities. Additional training and practice also occurred in regional small‐group settings, facilitated by the site leads. As the study progressed, these training and consultation sessions were also attended by the research assistants, who worked closely with the PPI members on site‐specific activities and interview data collection.

### 2.4 Planned PPI at different stages of the study

The core tasks for the PPI team, outlined at the start of the study, were to (i) support recruitment activity, (ii) conduct qualitative interviews with service users regarding their experiences of SlowMo therapy and (iii) support dissemination activity. The Sussex PPI lead (S. R.) contributed to the design of the evaluation, advising on the creation of the PPI log, sharing the GRIPP‐2 reporting tool and supporting the decision to report the PPI evaluation. Early PPI activity comprised consultation regarding recruitment materials and activities, and content of the qualitative interview topic guide. Subsequent input used a more formal collaborative PPI model. It involved PPI members acting as peer researchers to collect interview data, analyse sections of transcribed data, and co‐produce resulting themes from the qualitative substudy with the research team, as well as co‐producing the Plain English results summary, and providing written project summaries for use in lay journals and future publications.

### 2.5 Measurement of the impact of PPI

The impact of PPI on the project was captured in a number of ways. First, a PPI log in the form of an excel spreadsheet was created in consultation with the PPI team. This log enabled the PPI team to create a written record of (i) recommendations that arose from site and whole‐team meetings, (ii) the study team response to these recommendations, (iii) whether recommendations were implemented and (iv) the PPI team's perspective on the outcome. Recommendations were proposed by PPI members during PPI meetings, and recorded in the log by the PPI lead or research assistant. This log provided the opportunity for a quantitative record of the recommendations made and the percentage of these that were adopted. Second, at various stages throughout the project, both early in relation to consultation and later during the qualitative substudy, the PPI team provided written feedback on their qualitative subjective experiences of involvement. Third, PPI members attended study meetings and their impact was documented in meeting minutes. Finally, there were tangible impacts in the form of observable outputs produced by and as a result of the PPI group. Factors that enabled or hindered PPI, comprised reflections over the course of the evaluation from the PPI leads.

## 3 RESULTS

### 3.1 Measurement of PPI impact

The PPI team made substantial contributions to the SlowMo study across all phases of the study, as captured through the measurement of PPI impact. First, the PPI log (see Table 1) revealed a total of 107 actions or recommendations arising out of the PPI meetings, of which 87 (81%) were acted on. A number of actions were proposed that emerged out of the PPI discussions, that were not part of, or went beyond that which was initially expected from the PPI team. These actions are included in the actions recommended and acted on in the table, but examples are also listed in the footnote to Table 1, and in the section on wider impacts below.

**Table 1:** Log of involvement recommendations and outcomes

	Site		
Recommendation	Sussex	Oxford	London	Total (% acted on)
Recommendations regarding recruitment	9	3	4	16
Acted on	7	2	4	13 (81%)^a^
Recommendations regarding interviews	9	4	4	17
Acted on	9	3	4	16 (94%)^a^
Recommendations for dissemination^b^	13	0	4	17
Acted on	8	0	2 (2 uncertain)	10 (59%)^a^
Emergent novel recommendations^c^	12	3	0	15
Acted on	9	0 (1 uncertain)	0	9 (60%)^a^
Organizational recommendations	37	2	3	42
Acted on	35	1 (1 uncertain)	3	39 (93%)^a^

Abbreviation: PPI, Patient and Public Involvement.

^a^ Recommendations not acted on included—(a) in Recruitment—recruitment via NSUN (National Survivor User Network); presentations to peer support groups; use of SlowMo hashtags on twitter for wider recruitment. (b) Interviews—interviews conducted also in the TAU arm. (c) Dissemination—use of Twibbons; thunderclaps on twitter; a SlowMo facebook site; a mission statement on the SlowMo website page; a twitter session by the PPI team; an evaluation of the long‐term effects on social media of the BBC One Show; and written research assistant feedback about their own experience of working with PPI team. (d) Emergent recommendations—to monitor outcomes for people who had completed a related study; training of an additional therapist so that SlowMo could continue in a site when the trial stopped (although this is part of the next implementation phase); the establishment of peer support such as a SlowMo recovery college after the end of therapy; professional photos, and stories from the public, for the SlowMo people website; and a function whereby members of the public could submit their stories to the SLowMo people website. (e) Organizational—PPI members to join central study meetings remotely via skype.

^b^ Dissemination included social media dissemination, PPI reports and testimonials to team meetings, conference presentations, book chapters, and contributions to the website and Plain English summary.

^c^ Emergent novel recommendations included the SlowMo people webpage; service user interview, video and BBC One Show film; letter regarding the importance of PPI; and Publication on PPI impact in SlowMo.

Second, qualitative feedback from the PPI team revealed impacts for the study, the PPI participants themselves and the NHS more widely, the details of which are summarized in Figure 2.

Figure 2Service user consultant and peer researcher's written subjective experiences of Patient and Public Involvement in the SlowMo study

**Figure:**
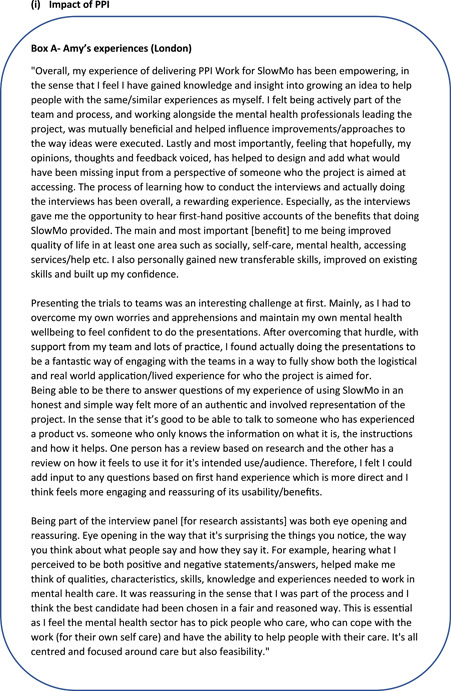

**Figure:**
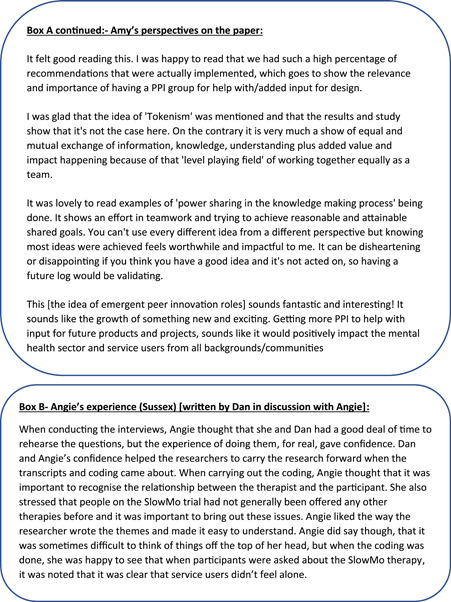

**Figure:**
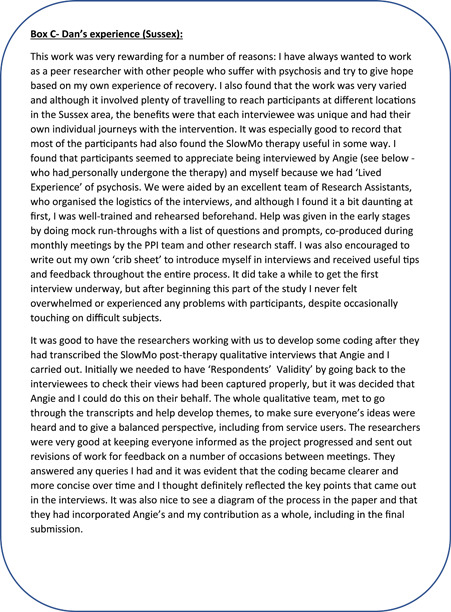

Third, minutes from study management meetings indicated that direct attendance and input of PPI members to these meetings occurred annually, despite an initial plan for at least 6‐monthly attendance. In addition, one PPI member attended the launch meeting in 2018, two PPI members attended a study management meeting in 2018 and three members attended a study management meeting in 2019 that had a specific PPI focus and presentation.

Finally, there were numerous tangible products from the PPI input, the impact of which are outlined below, such as the PPI team conducting service user interviews, producing additional recruitment leaflets and providing interviews for the local press, leading to the BBC film and coverage that significantly enhanced the research impact.

### 3.2 The impact of PPI

#### 3.2.1 On the research

The positive impacts of PPI on the research study included the production of new recruitment leaflets and attendance at community team meetings to promote recruitment, the collection and analysis of interview data to explore service users' experience of SlowMo therapy and the co‐production of the Plain English summary. The leaflet was produced by the team as additional patient‐facing information to aid recruitment. It was also used by the PPI team as promotional material, at team presentations, recovery college meetings and other such groups where service users were in attendance. For the qualitative interview study, the PPI team co‐produced the focus group topic guide and together conducted 22 qualitative interviews, all of which were led by either two PPI members or a PPI member supported by a research assistant. The data were analysed in two phases. In the first phase, the Sussex PPI team analysed a transcript collaboratively to produce a coding framework and held a series of meetings to reach consensus on initial themes. In the second phase, feedback was obtained through consultation with the London and Oxford PPI teams, and a further set of meetings led to a consensus on the final themes. In terms of study management, the PPI team co‐produced regular summaries of PPI input to the steering committee and funding body. Dissemination outputs to date have included the co‐produced Plain English summary as well as a paper on the qualitative substudy of service user experiences of SlowMo therapy.^22^ The PPI paper itself was written by the lead author, in collaboration with PPI members who reviewed and revised the content and provided the PPI narratives.

The major benefits of the PPI on the trial were that the target recruitment was achieved with support from the PPI team, the qualitative substudy was co‐produced and provided rich data concerning service users' experiences of SlowMo therapy and the Plain English summary of the results was co‐produced: PPI members were provided with key method and results sections, and were encouraged to write a lay summary in their own words. This was then drafted, shared and final feedback was obtained before completion to produce the Plain English Summary. A further impact of the PPI input lay in the emergent outcomes, which will be described under wider community impact.

#### 3.2.2 On the individual PPI members

There was a consistent core PPI group of five members throughout the whole 3‐year study. PPI members worked well together and became more confident in their roles over time. Subjective qualitative feedback (see Figure 2) revealed that PPI members felt that the PPI was well organized. Even though it was daunting and challenging, involved a lot of travel, involved overcoming the hurdle of team presentations, and was difficult to think on the spot, PPI members felt well informed, well trained and encouraged, and were given time to understand and contribute, such that everyone's ideas were heard. PPI members reported impacts on their confidence, career aspirations, knowledge, insight and skills to support themselves in their roles. They felt that the work was varied, rewarding and empowering. No problems were identified, even though difficult topics were discussed, and the PPI input was valued, including by service user participants who appreciated being interviewed by service users who understood them. They felt that there was mutual benefit in helping to improve how ideas were executed and in providing authentic first‐hand experience from someone the therapy is aimed at. However, not all PPI members provided subjective feedback, and this was provided by PPI members who were more actively engaged.

#### 3.2.3 Wider impacts

Importantly, in addition to the expected activities and resulting impacts, there were a variety of linked and ‘emergent’ activities and impacts. At a regional level, one of the PPI members produced an open letter reflecting his positive experiences of PPI membership and the importance of both PPI and research, alongside front‐line NHS work. This letter was used by the regional NHS PPI lead, to promote and encourage more service users to take up PPI roles.

The PPI team developed the concept of the ‘SlowMo People’ webpages (see http://slowmotherapy.co.uk/slowmopeople/). Based on the Humans of New York website, it aimed to tell individual stories of how fast thinking can trip you up, and how slowing down for a moment can be helpful. Drawing on both service user and researcher stories, the aim was to normalize the fast thinking style whilst also presenting real‐life personal experiences of the impact of slowing down.

Finally, the Sussex PPI lead, team and therapist worked with one PPI member, the NHS communications team and a local newspaper to produce an article about the experience of paranoia and voice hearing and the positive impact of receiving the SlowMo intervention. This was picked up nationally, resulting in a short film that was aired on prime‐time television on the BBC One show in April 2018 (see http://slowmotherapy.co.uk/news-2/). The BBC One show has an audience of 5 million people. Feedback via twitter suggested that the film had a major community value in providing a normalizing portrayal and hopeful outcome for psychosis and voice hearing, both for the general public, and for people across the country who are suffering with these experiences. The short film was also subsequently shown to people at the start of the therapy.

### 3.3 Factors that enabled or hindered the process or impact

Enablers to PPI included leadership expertise, localized groups, prioritized meetings, membership stability, individual support plans and genuine willingness of the research team to engage with the PPI process. The challenges included geography, travel, funding, regional PPI co‐ordination and well‐being of the PPI team.

#### 3.3.1 Contextual factors that enabled or hindered the process or impact

The PPI plan was led by the lead author, who has significant experience in co‐leading PPI work from the Sussex site, which itself has a good PPI track record. The study ran across three sites, and an early decision was made to hold a small PPI group at each site to contribute meaningfully to local recruitment challenges, and enable interview data collection. PPI was also specifically prioritized for collaborative discussion at a small number of study management meetings that were well planned, and co‐ordinated in advance, to enable PPI attendance.

However, PPI should ideally be led or co‐led by peer researchers, and support was subsequently enlisted from Sussex Partnership PPI leads to facilitate delivery. PPI co‐ordination was led from one site (Sussex), and other sites had more limited capacity to co‐ordinate local PPI groups, given different staffing and other challenges. In addition, several PPI members struggled with travel. As a result, PPI meetings at one site were less frequent, and preparation and conduct of qualitative interviews were more challenging, with fewer interviews conducted. The study management meetings were in the central site (London), which involved significant time and travel, and again created challenges for some PPI members in attending meetings. There were also variations in PPI members' confidence and capacity to use technology to join meetings remotely. As a result, PPI team members' input to study management meetings was limited, although it was represented as a standing agenda item at each meeting with written reports from the PPI team or verbal reports provided by the lead author.

#### 3.3.2 Process factors that enabled or hindered impact

The study team welcomed PPI involvement in the study and responded creatively and flexibly to ideas and challenges as they arose. The PPI team remained relatively stable, with five PPI members contributing for the entire project. Flexible individual support plans were put in place to enable meaningful contributions from all members despite fluctuations in well‐being. An agreement was reached to fund costed service user consultants' time beyond the end of funding for specific dissemination activities.

However, funding was comparatively limited for PPI co‐ordination and input at this multisite level. This may have affected the robustness of data collection for the PPI log, although redistribution of funds across sites based on activity level ameliorated other impacts. There was a potential challenge with respect to the aim for meaningful PPI input to dissemination, as these activities occurred beyond the funding window. These included the drafting of the Plain English summary, other dissemination materials, the qualitative project publication, website updates and presentation at the stakeholder event. There was variation in attendance at the whole‐site PPI training and consultation sessions, related to factors such as mental well‐being. Finally, there were understandable fluctuations in the life circumstances, health and well‐being of PPI members in all sites, which impacted participation in meetings and other PPI activities. Three PPI members stopped attending PPI meetings before the end of the study: one after a period of illness; one due to workload on other projects; and a third was not contactable by their original phone number towards the end of the study. A fourth PPI member sadly passed away. Naturally, this had a significant impact on the team and was discussed both individually within the local site and as a wider team in the subsequent all‐site meeting.

## 4 DISCUSSION

### 4.1 How PPI influenced the whole study

The main impacts of PPI within and beyond the SlowMo trial were within the qualitative substudy^22^ and the emergent innovations that were part of the wider community impacts. The qualitative substudy was a planned part of the research project, which aimed to investigate participants' experiences of the SlowMo intervention, and the associated blended digital approach, including use of the in‐session webapp and mobile phone app. This was a strength of the planned PPI input as the substudy was fully collaborative from the development of the topic guide, to the PPI in collection of all interviews across all three sites, to the whole PPI group involvement in the thematic analysis and the final summary of results. This substudy is reported in detail in a separate paper.^22^ The contribution of PPI to the recruitment of participants in one site was highly impactful and completely opened up responses to the study from some teams, where referrals went from zero across two different trials to recruitment of 15–20 people from the same team. PPI was also central to the recruitment of excellent research staff, with a PPI member playing an active role on the interview panel. The most significant emergent innovations included the BBC One Show broadcast and SlowMo people webpages, which, although consistent with planned dissemination activity, were highly innovative and creative, and went well beyond the initial expectations of producing patient leaflets, lay summaries, presentations, co‐produced peer‐reviewed journal articles and papers in service user journals.

### 4.2 Limitations of the PPI contribution in the SlowMo study

Overall, the PPI contribution to the SlowMo study was well supported, with clear impacts on the research and wider society and positive experiences for individual PPI members, who felt valued, supported, empowered, rewarded and understood and that their contributions mattered. PPI members also described personal growth in knowledge, skills and confidence. The PPI in the SlowMo study met five of the six UK standards for PPI (2020)^23^ in being inclusive, working together, supporting learning, employing plain‐language communication and evaluating impact. The only standard not explicitly met related to involvement in research governance, which was less relevant to the specific project. However, whilst many aspects of PPI in the SlowMo study went exceptionally well, there were several challenges. The funding requested for PPI was lower than INVOLVE recommendations^23^; there was initially no service user PPI co‐lead; and there were challenges to managing PPI across geographies and sites. In future, it would be valuable to include PPI at managerial levels and evaluate the impact on recruitment, retention and stigma reduction. Whilst some peer‐led suggestions and innovations were adopted, others were only partially taken forward or were not supported due to the lack of capacity within the PPI and research teams, or the need to deliver specific a priori trial objectives.

### 4.3 The evaluation of PPI impact in the SlowMo study

There are many advocates of the need to evaluate the impact of PPI in research.^24^, ^25^ Yet, a common criticism of PPI is that it is difficult to demonstrate its unique contribution and added value to a research project. Some studies have evaluated PPI systematically using questionnaires and semistructured and qualitative interviews repeated longitudinally.^26^ However, this approach may in and of itself be couched in empirical research methodology.^27^, ^28^ Indeed, Friesen et al.^21^ have argued that involvement is more than might be captured by the singular epistemic focus on research impact.^29^

In the SlowMo study, we planned to evaluate the impact both quantitatively in terms of the proportion of PPI recommendations that were adopted of those that were recorded in the log and qualitatively in terms of subjective feedback and study group document review. The log was relatively well maintained, but due to resourcing issues and challenges of updating across multiple sites, it is possible that some more minor entries were omitted. It is also acknowledged that the proportion of recommendations that were adopted is a blunt measure of impact, being dependent on the number and nature of recommendations made and the ease with which they could be achieved. Some recommendations had greater potential impact and value than others, and a future log might also consider the nature and relative weight of the recommendations adopted and the reasons for them not being enacted. Subjective qualitative experiences were limited to PPI members who were more engaged, thus being open to the criticism levied by Petit‐Zeman and Locock^30^ that perhaps diverse voices were not being heard. The PPI team collaborated on and were heavily involved in the qualitative substudy of service user experiences of the SlowMo intervention. This study produced new knowledge in the form of a richer understanding of service user experiences of the trial, the intervention content, the blended therapy approach, service user recommendations to improve the technological experience and the contribution to outcomes. However, it could be argued that the plan for this substudy was developed by the research team and that while the co‐production was really strong, the added value of the PPI collaboration could not be fully disentangled.

Perhaps, the clearest and most tangible impacts were not those that emerged from the narrow epistemic focus on enhanced research quality, but those that arose as unique outcomes with added value from the PPI, such as the BBC One Show film and the SlowMo people webpage. There is often limited scope for these emergent community‐based impacts within a funded research study, and several other such innovations such as the use of thunderclaps, twibbons and a public facebook page that were also proposed by the PPI team were not taken forward. Whilst a variety of factors affected these decisions, funded research studies may necessarily be forced to limit unanticipated innovation.

### 4.4 Theoretical–conceptual developments in the definition of PPI roles

Traditional PPI roles utilized in SlowMo included the critical friend model of consultation, and the peer researcher model of collaboration. These roles impacted on the design, ethics and delivery of the research as well as on participants, researchers, PPI members, organizations and the wider community.^5^, ^8^ However, as highlighted by Friesen, PPI should focus not only on the impact of PPI on research knowledge, but also on the way in which power and decision‐making are shared in the knowledge‐making process.^21^

We propose that an important and novel role for PPI in research is that of emergent ‘Peer Innovator’. Experience within the SlowMo study and other studies^13^, ^14^ has identified that an added value of PPI in research is the unexpected, emergent outcomes that arise when a group of enthusiastic service users come together within a collaborative framework linked to a specific study. There is significant potential for impact, arising from the freedom and desire to extend this impact to aid service users and communities beyond the specific predesignated constraints of the research study. In the current study, there were numerous emergent ideas and outcomes, including the newspaper article and BBC One Show coverage, and the SlowMo people webpage. By taking these ideas forward, the SlowMo PPI collaboration enabled power sharing in the knowledge‐making process, as recommended by Friesen et al.,^21^ to produce a response to community‐level ignorance and stigma: the BBC One Show being aired on prime‐time UK TV to over 5 million viewers. These ideas have the potential for widespread impact, but not all can be supported within a specific research study and budget. A challenge for future PPI in research will be how to ensure cost‐effective study delivery whilst providing space and support for peer innovation where it emerges. Our PPI team (Figure 2) emphasized the opportunities with this role, which was seen as ‘fantastic and interesting! The growth of something new and exciting’. Getting more PPI to help with input for future products and projects would positively impact the mental health sector and service users from all backgrounds/communities.

### 4.5 Future recommendations

Future projects would benefit from a requirement for a comprehensive PPI plan, alongside the detailed project plan, at the grant application stage, costed with reference to INVOLVE guidance.^23^ We propose that a proportion of the PPI plan be permitted to be allocated to support emergent peer innovation, to allow for the development of important creative products and impacts that arise from this PPI collaboration. The enhanced community impact and higher national profile for PPI roles arising from peer innovation, might encourage more service users to take up this role. This would in turn create a larger and more diverse pool of peer researchers from which PPI leads would emerge. PPI members should include a representative balance of genders, ethnicities and engagement experiences. To harness creativity and ensure diversity of representation, will require increased flexibility of opportunities for engagement, and proactive outreach.

## CONFLICT OF INTEREST

Prof. Freeman reported receiving personal fees from Oxford VR outside of the submitted work. No other disclosures were reported.

## AUTHOR CONTRIBUTIONS

Prof. Kathryn Greenwood had full access to all the data in the study and takes responsibility for the integrity of the data and the analysis. *PPI plan*: Prof. Kathryn Greenwood, Prof. Philippa Garety, Drs. Thomas Ward, Amy Hardy and Sam Robertson. *Conduct of the PPI plan, acquisition, analysis or interpretation of data*: Prof. Kathryn Greenwood, Prof. Philippa Garety, Drs. Thomas Ward, Amy Hardy, Sam Robertson, Alison McGourty, Cat Sacadura, Mar Rus‐Calafell, Nicola Collett as well as Evelin Vogel, Claire Vella and the SlowMo PPI team. *Drafting of the manuscript*: Prof. Kathryn Greenwood and SlowMo PPI team members. *Critical revision of the manuscript for important intellectual content*: All authors including SlowMo PPI team members.

## Data Availability

The data that support the findings of this study are available on request from the corresponding author. The data are not publicly available due to privacy or ethical restrictions.
